# Improving the Positive Predictive Value of Non-Invasive Prenatal Screening (NIPS)

**DOI:** 10.1371/journal.pone.0167130

**Published:** 2017-03-01

**Authors:** Charles M. Strom, Ben Anderson, David Tsao, Ke Zhang, Yan Liu, Kayla Livingston, Christopher Elzinga, Matthew Evans, Quoclinh Nguyen, David Wolfson, Charles Rowland, Paula Kolacki, Megan Maxwell, Jia-Chi Wang, Douglas Rabin, Joseph Catanese, Renius Owen, Corey Braastad, Weimin Sun

**Affiliations:** 1 Quest Diagnostics Nichols Institute, San Juan Capistrano, California, United States of America; 2 Athena Diagnostics, Marlborough, Massachusetts, United States of America; 3 Celera Diagnostics, Alameda, California, United States of America; National Cheng Kung University College of Medicine, TAIWAN

## Abstract

We evaluated performance characteristics of a laboratory-developed, non-invasive prenatal screening (NIPS) assay for fetal aneuploidies. This assay employs massively parallel shotgun sequencing with full automation. GC sequencing bias correction and statistical smoothing were performed to enhance discrimination of affected and unaffected pregnancies. Maternal plasma samples from pregnancies with known aneuploidy status were used for assay development, verification, and validation. Assay verification studies using 2,085 known samples (1873 unaffected, 69 trisomy 21, 20 trisomy 18, 17 trisomy 13) demonstrated complete discrimination between autosomal trisomy (Z scores >8) and unaffected (Z scores <4) singleton pregnancies. A validation study using 552 known samples (21 trisomy 21, 10 trisomy 18, 1 trisomy 13) confirmed complete discrimination. Twin pregnancies showed similar results. Follow-up of abnormal results from the first 10,000 clinical samples demonstrated PPVs of 98% (41/42) for trisomy 21, 92% (23/25) for trisomy 18, and 69% (9/13) for trisomy 13. Adjustment for causes of false-positive results identified during clinical testing (eg, maternal duplications) improved PPVs to 100% for trisomy 21 and 96% for trisomy 18. This NIPS test demonstrates excellent discrimination between trisomic and unaffected pregnancies. The PPVs obtained in initial clinical testing are substantially higher than previously reported NIPS methods.

## Introduction

Noninvasive prenatal screening (NIPS) using cell-free DNA (cfDNA) from maternal plasma is an effective option for detecting trisomy 21, trisomy 18, and trisomy 13 in pregnancies at high risk for fetal aneuploidies. NIPS provides higher detection rates than traditional methods such as maternal serum screening and nuchal translucency testing. NIPS can also reduce the rate of false-positive results [1–4] and thus the number of unnecessary invasive procedures, which carry a risk of procedure-related miscarriage (roughly 0.5% to 1.0% [5]) and other complications [6].

The great potential of NIPS has led to the development of multiple NIPS tests. Assays that use single-nucleotide polymorphism (SNP)-based methods [7], chromosome-specific sequencing (CSS) [8,9], or massive parallel shotgun sequencing (MPSS) [10–12] are commercially available. Although performance can vary slightly by assay [8,9,13–16], the generally high sensitivity and specificity [17] have led multiple professional societies to recommend that NIPS be offered as a screening option for fetal chromosome aneuploidies in high-risk pregnancies [6,17,18]. Guidelines emphasize the importance of counseling patients when NIPS testing is considered and stipulate that positive NIPS results must be confirmed by diagnostic tests [6,17,18]. These recommendations recognize that, although NIPS lowers the risk of false-positive results compared to conventional methods, it does not eliminate them altogether.

NIPS false-positive rates affect assay specificity as well as the positive predictive value (PPV). The PPV of any test is proportional to its specificity and the prevalence of the disorder. For example, a test with 100% sensitivity and 99% specificity for a disorder with a prevalence of 1:100 (1%) will have a PPV of only 50%, since for every 100 tests there will be approximately 1 true-positive and 1 false-positive result. Because the prevalences of trisomies 21 (1:185), 18 (1:470), and 13 (1:1500) [19] are low even in high-risk populations, even a test with 99.9% specificity (false-positive rate of 0.1%) would yield PPVs of 90% for trisomy 21, 67% for trisomy 18, and 53% for trisomy 13. In fact, these PPVs are close to those observed in a study of 109 prenatal specimens analyzed in our clinical reference laboratory [20]. In that study, CVS and amniocentesis specimens submitted for cytogenetic evaluation after NIPS were analyzed for aneuploidies. The resulting PPVs of NIPS were 93% for trisomy 21, 64% for trisomy 18, and 44% for trisomy 13. We expanded this original study to include 211 consecutive specimens, along with combined data from more recent publications, for a total of 1547 samples in the combined data set (Table 1). The cumulative PPVs were 91% for trisomy 21, 73% for trisomy 18, 39% for trisomy 13, and 49% for sex chromosome aneuploidies. The similarity of these numbers to our original study suggests that the PPV is not improving over time for the previously available first-generation NIPS tests. Meck and colleagues recently reported similar PPV results in a series of 216 samples referred for invasive testing following NIPS [19]. To improve the PPV of NIPS for aneuploidies, the false-positive rate must be further decreased.

**Table 1:** Positive Predictive Values for Noninvasive Prenatal Screening Performed at Third-party Laboratories

NIPS Result (prevalence)	Current Study^a^, Number Cases (PPV)	Current Study + Literature [19,30–34] Number Cases (PPV)
Trisomy 21 (1:185)	84 (85%)	1174 (91%)
Trisomy 18 (1:470)	53 (57%)	350 (73%)
Trisomy 13 (1:1500)	28 (36%)	136 (39%)
Sex Aneuploidy (1:1000)	39 (38%)	115 (49%)
Microdeletions (3:000)	13 (38%)	Not Determined

^a^ Based on results of invasive follow-up testing performed at Quest Diagnostics; NIPS performed elsewhere. The performing laboratory was known in 86 samples and included Natera (43 samples), Sequenom (20) [30], Ariosa (16), and Verinata (7).

NIPS false-positive results can be caused by biological factors (eg, confined placental mosaicism, vanishing twin syndrome, fetal or maternal mosaicism, tumors, and maternal duplication), as well as technical issues. In a recent study, 2 of 4 pregnancies with discordant prenatal test results were caused by maternal duplication events on chromosome 18 [21]. Further analysis indicated that maternal duplication may be a substantial contributor to false-positive rates of NIPS [21]. The identification and elimination of false-positive results caused by maternal duplication or other factors may improve the PPV of NIPS. To achieve this, we designed an MPSS-based clinical NIPS assay for fetal chromosome aneuploidies. This laboratory-developed test (LDT) utilizes automation of all manual processes, a proprietary high-yield method of cfDNA preparation, automated library quantification and dilution, and “Version 4” chemistry (Illumina) for HiSeq next-generation sequencing reagents. In addition, it incorporates GC sequence bias correction and the use of proprietary bioinformatics and biostatistics processing to allow complete discrimination between affected and unaffected pregnancies. In this report, we present validation data and the initial clinical results of the test. We also demonstrate the contribution of maternal duplications and global copy number changes to false-positive results in a clinical laboratory setting.

## Materials and Methods

### Patient samples

For assay development, verification, and validation studies, we obtained samples from pregnant women from Sequenom, Precision Medicine, and consented volunteers. For singleton pregnancies we obtained 3,750 samples from Sequenom, 165 from Precision Medicine, and 10 from volunteers; Sequenom also provided samples from 115 twin gestations. The Sequenom samples were scheduled to be discarded and were de-identified before being sent to us. The samples from Precision Medicine were consented using their protocols. Volunteers provided written informed consent via signed forms approved by the Western Institutional Review Board, which specifically reviewed and approved this study. The study was conducted according to the principles in the Declaration of Helsinki.

### Next-generation sequencing

Whole blood was collected in two 10 mL Cell-Free DNA BCT blood collection tubes (Streck, Omaha, NE) and transported at room temperature. Blood tubes were processed within 4 days of draw. The plasma was isolated from each of these samples using a Tecan EVO 200 liquid handler (Tecan, Männedorf, Switzerland). The Tecan EVO 200 liquid handler performs the following activities: centrifuges the Streck blood tubes at 22°C for 10 minutes at 2,500 x g, transfers the plasma to a 15 mL conical tube, centrifuges the 15 mL conical tube at 22°C for 20 minutes at 3,200 x g, and transfers plasma to a final 15 mL conical tube. The cell-free DNA (cfDNA) is then extracted from 4 mL of plasma using DynaMax chemistry (Thermo Fisher Scientific, Waltham, MA), following manufacturers recommendations, with the aid of a Kingfisher Flex Purification System (Thermo Fisher Scientific). cfDNA was made into sequencing ready libraries using the NEBNext^®^ Ultra^™^ DNA Library Prep Kit for Illumina^®^ (New England BioLabs Inc, Ipswich, MA) following the manufacturer’s recommendations. During PCR, a 10-bp barcode is amplified onto each sample using the reverse PCR primer,. All reactions shared a common forward primer. The universal forward primer sequence was: AATGATACGGCGACCACCGAGATCTACACTCTTTCCCTACACGACGCTCTTCCGATCT; the reverse primer was: CAAGCAGAAGACGGCATACGAGATXXXXXXXXXXGTGACTGGAGTTCAGACGTGTGCTCTTCCGATCT, where X denotes the 10 base barcode location. PCR was performed on a SimpliAmp Thermal Cycler (Thermo Fisher Scientific). PCR conditions were as follows: initial denaturation at 98°C for 30 seconds, 10 cycles of denaturation at 98°C for 10 seconds, annealing at 65°C for 30 seconds and extension at 72°C for 30 seconds, final extension at 72°C for 5 minutes; the amplification ends with a 4°C hold. Following PCR, the products were purified using the Agencourt AMPure XP PCR Purification beads (Beckman Coulter, Brea, CA) following the manufacturer’s recommendations. The AMPure bead-to-PCR product ratio was 1:1. The cleaned-up PCR products were quantified using the Quant-It PicoGreen dsNDA Assay Kit (Thermo Fisher Scientific), following the manufacturer’s recommendations, and read on an Infinate 200 PRO Microplate Reader (Tecan). Samples were normalized to 2 nM and pooled with 12 samples in each library. Library pools were denatured and further diluted to 15 pM. A 5% PhiX Control (Illumina, San Diego, CA) was spiked into each pool. The pooled libraries were clonally amplified and bound to high-output flow cells (Illumina) using the cBot system from Illumina. Sequencing was performed on a HiSeq2500 system by single read 36 cycles followed by 10 cycles to sequence the index. A minimum of 9 million reads were required for the bioinformatics process. Data were streamed from the HiSeq2500 system to an Isilon (EMC Isilon, Seattle, WA) server, where the data analysis pipeline was begun automatically.

We used a read length of 36 base pairs in one direction at an average sequencing depth of 0.6X. All quality score “Q scores” were > 30.

Bioinformatics and statistical calculations were performed using a proprietary analysis pipeline; a detailed description of this pipeline is beyond the scope of this article. In brief, aligned reads are mapped to “bins” throughout the genome. The bins have been carefully chosen to contain sequences unique to that chromosomal region. A Z-score is calculated for each bin and then averaged across the entire chromosome to obtain a chromosome-specific Z score. Because the Z score is a multiple of the standard deviation of the assay, assay performance depends on the consistency of the assay. The more consistent the assay, the greater the separation between affected and unaffected pregnancies.

Chromosomes 13 and 18 are GC-rich relative to other chromosomes, causing sequencing bias that can skew the percentage of counts mapped to those chromosomes. We therefore used a published R-script for GC correction [22], with further statistical smoothing using a proprietary algorithm. The means and standard deviations were established for chromosomes 13, 18, 21, and X following GC correction. A more detailed description of the bioinformatics calculations appears below.

### Bin read count data normalization, GC correction, chromosome representation and Z score calculations

As reported [22], bin read count data were first scaled by its own sample autosomal total read counts and then GC correction was performed using the local polynomial regression fitting R loess function and hg19 data. A PCA model was applied to such normalized data to remove high-order artifacts. As reported [23,24], chromosome representations were calculated as

$$chrRep_{i}\;=\;\frac{chrTotalRC_{i}}{\sum_{j=1\ldots 22}chrTotalRC_{j}}$$

and each chromosome Z score was calculated as

$$Z\;=\;\;\frac{x\;-\;\mu }{\sigma }$$

*x*: sample chromosome representation

μ: chromosome representation plate median

σ: chromosome representation MADs, median absolute deviation, as calculated using a training set of 5406 samples.

### Sex chromosome aneuploidy detection

#### Analytical method

Read counts within 50 kilobase bins were used as the input for the sex chromosome aneuploidy analysis. Sample results having read counts lower than 4.5 million were excluded from the analysis. Read counts for bins within the X and Y chromosomes were normalized for each sample using autosomal read counts.

#### X-chromosome-based fetal fraction estimation

After gender is determined using established absolute Y-chromosome read count thresholds, fetal fraction estimates using the X chromosome were calculated using the expected relationship between fetal fraction (*FF*) and X-chromosome representation.

$$FF\;=\;\;2\;\times \;\left(1\;-\;\frac{X_{i}}{X_{f}}\right),$$

where *FF* is the sample’s X-based fetal fraction estimate to be calculated, *X*_*i*_ is the median X-chromosome representation (median count within bin, normalized by autosomal count total), and *X*_*f*_ is the median of all median X-chromosome representation values for female samples on the run.

#### Reference file generation and parameter determination for Y chromosome estimates

After dividing the Y chromosome into 50 kilobase bins, well-performing chromosomal bins on the Y chromosome were determined by comparing training data of adult males and adult female samples (including non-pregnant females and samples from females pregnant with a fetal female). Y-chromosome bins having a mean adult male read count greater than fivefold the mean adult female read count, and having a mean adult male read count greater than 150 reads, were marked as well-performing bins on the Y chromosome.

The background (female) level of normalized Y-chromosome read counts and the slope of the relationship between normalized Y-chromosome read counts and male fetal fraction were estimated using previously established X-chromosome based fetal fraction estimates from a 3589-sample pool as training data. The established background level and normalized Y bin count slope estimates were used to calculate a Y-based fetal fraction estimate for each sample.

#### Sex chromosome aneuploidy detection

Training data from 649 samples were used to establish boundaries between normal samples and those positive for sex chromosome aneuploidies, by inspection of the X-chromosome-based FF estimates versus the Y-chromosome-based FF estimates (graph and thresholds below). Established test thresholds were then validated using 590 previously characterized samples.

Microduplications and deletions were calculated in a similar manner to whole chromosome trisomies. The only difference is chromosome trisomy with whole chromosome as a unit vs. MD, with predefined regions of a chromosome as a unit with predefined locations/coordinates on chromosomes utilized for microdeletions such as 1p36 or DiGorge. Total read counts were calculated as a summation of total read counts of bins within these locations/coordinates of chromosome.

### Fetal fraction estimations

**Fig 1:**
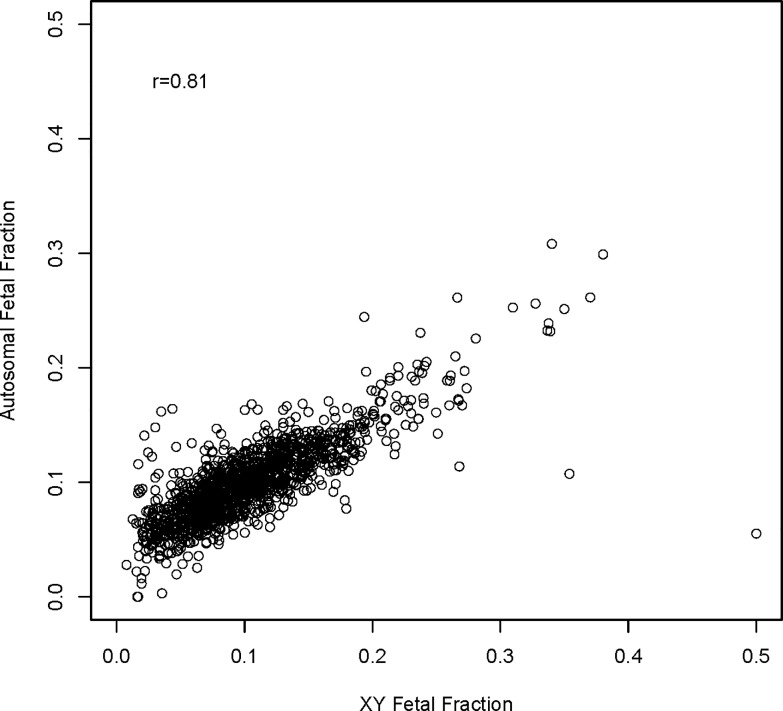
Relationship of fetal fraction estimated using a median of X and Y based methods with the fetal fraction estimated from a model of autosomal read counts among 1366 male samples that were not included in the development of the autosomal model. Pearson correlation coefficient (r) = 0.81.

Fetal fractions (FFs) were calculated based on X chromosome underrepresentation or Y chromosome overrepresentation using the following methods:

a) As reported [25], fetal fraction was estimated as $2\;\times \;(1-{\bar{N}}_{23}/\bar{N})$, where ${\bar{N}}_{23}/\bar{N}$ is the average read count per bin for chromosome X normalized to the autosome bin average. b) As reported [26], we used R package RAPIDR based on X chromosome under representation to estimate male FF based on X chromosome under representation. c) FF was estimated based on X chromosome underrepresentation with non-pregnant female as two X chromosome copy reference, non-pregnant male as single X chromosome copy reference, and known FF samples as standard controls. d) FF was estimated based on Y chromosome over representation with non-pregnant female as Y chromosome absence (0% Y) reference, non-pregnant male as Y chromosome presence (100% Y) reference and known FF samples as standard controls. For better male FF estimation, the median value of these four calculations was used as our final male FF and such median of four FF is correlated very well with a set of known FF sample shaving R square = 0.9752 with y-intercept = 0.
  For female fetuses, fetal fraction was estimated using a regularized regression model [27]. Briefly, a training set of 3281 samples from known male fetuses was used to model fetal fraction (estimated as described above) as a function of sample bin counts normalized by the sample total read count but uncorrected for GC content. Bins residing on chromosomes 13, 18, 21, X or Y chromosomes were excluded from the modeling process. The model was a regularized linear regression model implemented with the R package “glmnet” (version 1.9–8). Ten-fold cross-validation using an alpha parameter of 1 was used to select the lambda parameter having the minimum cross-validated error for use in building the final model which is subsequently used to estimate fetal fraction for female fetuses. Fig 1 demonstrates the relationship of fetal fraction estimated using a median of X and Y based methods with the fetal fraction estimated from a model of autosomal read counts among 1366 male samples that were not included in the development of the autosomal model. The Pearson correlation coefficient (r) was 0.81.

Once the assay was fully automated, we tested a series of 1,288 frozen plasma samples from unaffected pregnancies. These data allowed us to identify bins that were representative of each chromosome and eliminate regions of shared or homologous sequence. We then applied GC correction to the raw data. We can also display the normalized bin counts using an ideogram for any individual chromosome or the entire genome. The ideogram resembles a microarray result, with each point representing a normalized count for a particular bin on a particular chromosome. Positive Z scores indicate duplications (trisomy, if involving an entire chromosome) and negative Z scores represent deletions (monosomy, if involving an entire chromosome). Before reporting any positive result, the affected chromosome and the entire genome are reviewed to eliminate the possibility of maternal abnormalities causing the elevated Z scores.

Fetal fractions were calculated for male fetuses using Y chromosome-specific sequences. For female fetuses we developed a proprietary bioinformatics approach (a description is beyond the scope of this publication). The fetal fraction calculation was not used to modify the Z-score calculations.

### Initial clinical data

We are obtaining follow-up information for every positive NIPS result obtained through clinical testing at our reference laboratory. One of our genetic counseling team contacts the referring physician to determine the outcome of the pregnancy.

## Results

Once the performance parameters of the assay were established, we tested a series of verification samples including known unaffected and known aneuploid pregnancies. This series of 2,085 samples included trisomy 21 (n = 69), trisomy 18 (n = 20), and trisomy 13 (n = 17). No unaffected pregnancy had a Z score >4 and no affected pregnancy had a Z score <8. Following assay verification we analyzed a validation set comprising 552 samples, including samples known to be positive for trisomy 21 (n = 21), trisomy 18 (n = 10), trisomy 13 (n = 1), and XO (n = 1). Once again, no unaffected pregnancy had a Z score >4 and no affected pregnancy had a Z score <8.

Since there was no difference in performance between the verification and validation studies, we combined the results for analysis. The effects of GC correction were least for chromosome 21, which has normal GC content, intermediate for chromosome 18, known for having an intermediate increase and GC content, and greatest for chromosome 13, which has the highest GC content (Fig 1). Using raw data, a Z score threshold of 4 yielded absolute discrimination between the 2,498 unaffected pregnancies and the 90 trisomy 21 samples; no unaffected pregnancy had a Z score >4, and no affected pregnancy had a Z score <8. However, GC correction improved discrimination for chromosomes 13 and 18: without GC correction, most trisomy 13 samples had Z scores less than 4; after GC correction, all trisomy 13 samples had Z scores well over 8. GC correction also allowed complete discrimination of trisomy 18 from unaffected pregnancies. Therefore, after GC correction and biostatistical smoothing, the assay provided 100% discrimination between affected and unaffected pregnancies (Fig 1, right panel) demonstrates the combination of GC correction with statistical smoothing, which further improves assay performance.

We also analyzed a series of 115 samples from twin gestations with known aneuploidy status as part of assay validation, including 10 trisomy 21, 4 trisomy 18, and 13 trisomy 13 samples. Following GC correction and smoothing, all samples with autosomal trisomies had Z scores >11 and all unaffected pregnancies had Z scores <4. Overall, discrimination was greater in twin than singleton samples (data not shown), even though most twins would be expected to be discordant for autosomal trisomies.

As a final validation for trisomy detection, we obtained samples from 100 consented volunteer pregnant women and split the samples between our laboratory and Sequenom. Results were concordant in all cases. This series had 99 unaffected and 1 sample predicted to be from a woman carrying a fetus with trisomy 21 by both laboratories.

To assess the accuracy of the NIPS assay for fetal sex determination, we tested 372 (188 male) samples over the course of 6 different assay setups. Fetal sex had been previously determined using the Sequenom Maternity21 Plus assay, but was not phenotypically confirmed. Our NIPS assay yielded concordant results in all but 1 sample, in which results indicated a male fetus when a female fetus was expected. Thus, overall accuracy was 99.7% (371/372). However, the fetal fraction for this sample (2.75%) was below the 5% threshold for reporting (not shown) and would have prompted a request for a new sample in clinical testing.

### Clinical implementation

We accept samples beginning at the 10^th^ gestational week. Greater than 90% of samples received are from between the 10^th^ and 15^th^ gestation week.

Based on the above validation and verification results, for clinical implementation we used a Z score cutoff of ≤4 for unaffected pregnancies and >8 for affected pregnancies. Z scores >4 but < 8 prompted further examination. Review of our first 10,000 clinical samples revealed abnormal NIPS results in 180 (1.8%) (Table 2). Overall positive rates were 1.0% for trisomy 21, 0.36% for trisomy 18, 0.21% for trisomy 13, and 0.17% for sex aneuploidies. One sample was positive for the DiGeorge microdeletion and 2 cases had 2 abnormalities. Of the first 10,713 samples tested, results could not be reported in 94 (0.88%); the cause was low fetal fraction in 63 cases (0.59%) and uninformative DNA pattern, failure to meet quality metrics, or other technical issues in 31 samples (0.29%).

**Table 2:** Follow-up of Clinical Samples Positive for Fetal Aneuploidies on Non-invasive Prenatal Screening

Positive NIPS Result	Number singleton (twin)	Confirmation of Positive NIPS Result		No Follow-up Testing			False +	Follow-up pending	Lost to follow-up	PPV, %	Adjusted PPV^a^, %
		Karyotype	U/S or physical exam	SAB (twins)	Follow-up ongoing	Pregnancy Terminated					
T21	99 (4)	37 (3)	1	7 (1)	26	11	1^b^	10	6	98	100
T18	35 (1)	14	9	1	4	0	2^c^	4	2	92	96
T13	20 (1)	7	2	2	2	0	4^d^	3	1	69	NA
45,X	9	3	3	0	0	0	1^e^	2	0	86	100
47,XXX	5	2	0	0	1	0	1	1	0	67	NA
47,XXY	2	1	0	0	1	0	0	0	0	100	NA
47,XYY	1	0	0	0	1	0	0	0	0	NA	NA
22q del	1	1	0	0	0	0	0	0	0	100	NA
T21 & 45,X	1	0	0	1	0	0	0	0	0	NA	NA
T21 & T13	1	0	0	0	0	0	0	1	0	NA	NA

^a^ PVV excluding false-positives reclassified as true negatives based on changes in reporting rules.

^b^ Re-evaluation of data showed multiple chromosome variations (see text)

^c^ Twin gestation with one twin having mass felt to be teratoma (see text)

^d^ 1 patient with significant fibroids

^e^ Maternal 45,X/46,XX

**Fig 2:**
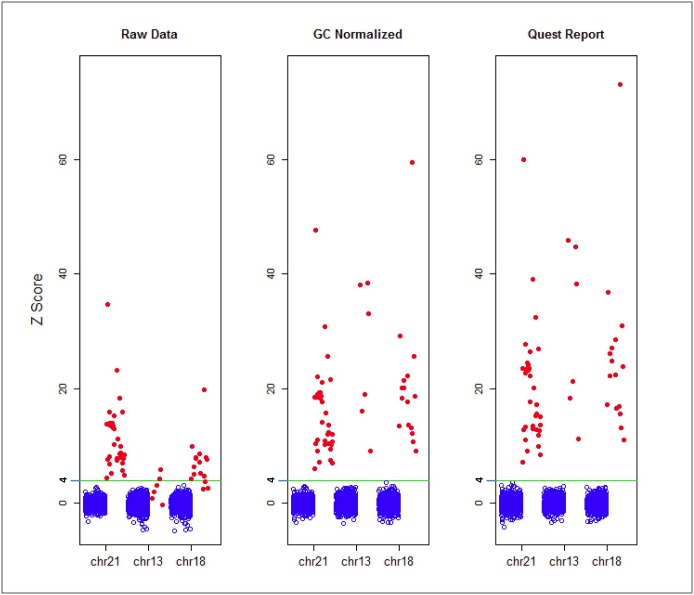
Z scores of the laboratory-developed noninvasive prenatal assay for trisomies 21, 18, and 13 before (raw) and after correction for GC content and statistical smoothing using a proprietary software algorithm. The assay provided complete discrimination between affected and unaffected pregnancies for trisomy 21, even without adjustments. GC correction and statistical smoothing eliminated the substantial overlap between affected and unaffected pregnancies for trisomies 18 and 13, and enhanced separation for trisomy 21.

**Fig 3:**
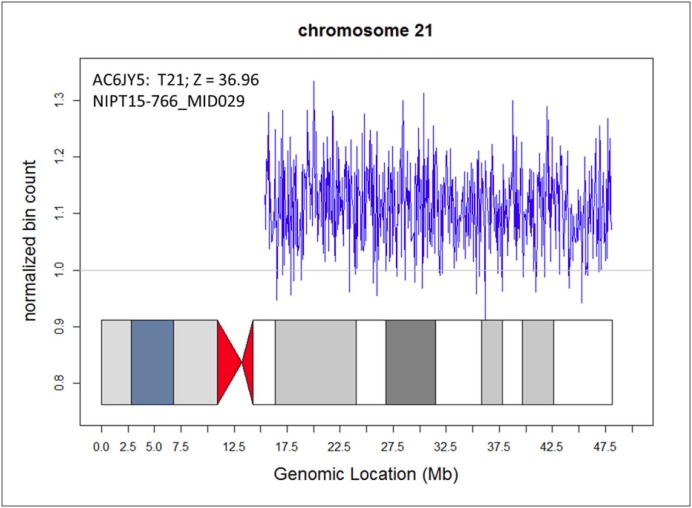
Ideogram for chromosome 21 from a prenatal sample positive for trisomy 21. Each point represents a normalized count for a particular bin on a particular chromosome; a euploid value on the Y axis is 1.0. As can been seen, the entire chromosome 21 demonstrated duplicated material. The Z score for this sample was 36.96.

**Fig 4:**
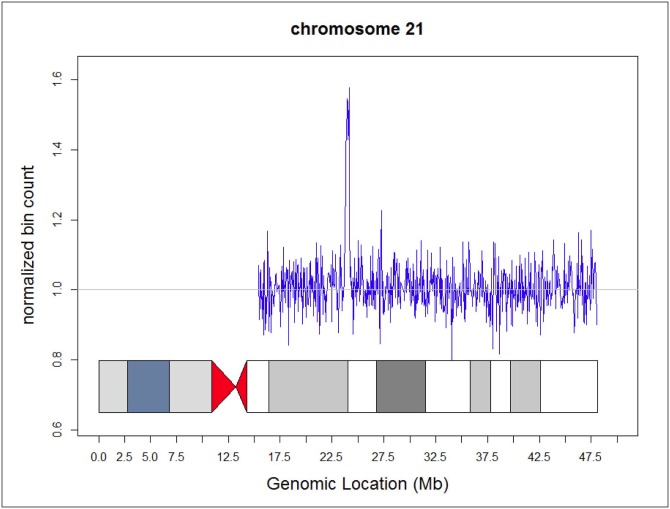
Ideogram for chromosome 21 from a patient with a maternal microduplication.

**Fig 5:**
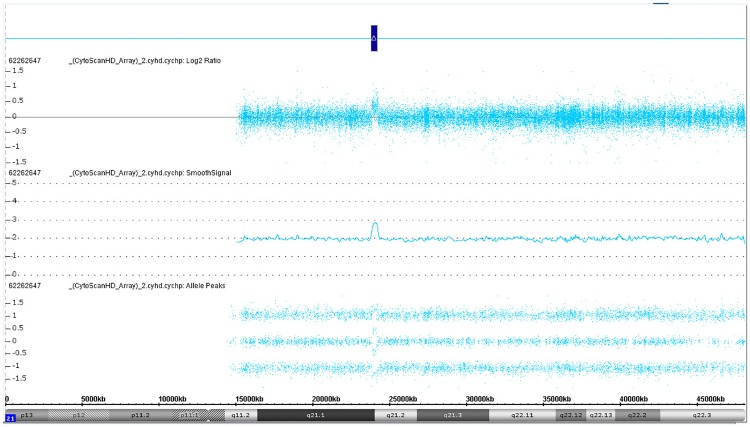
Microarray data for maternal DNA for the patient in Fig 4.

**Fig 6:**
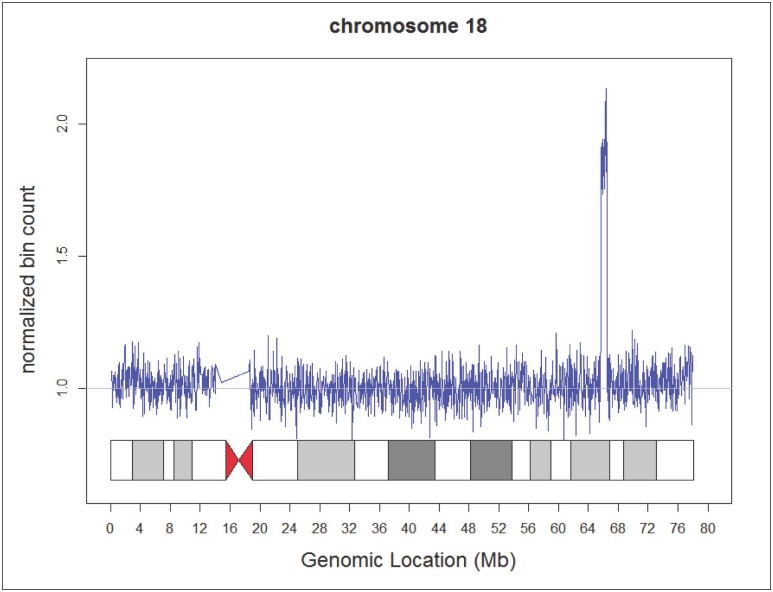
Ideogram for chromosome 18 from a patient with a maternal microduplication.

**Fig 7:**
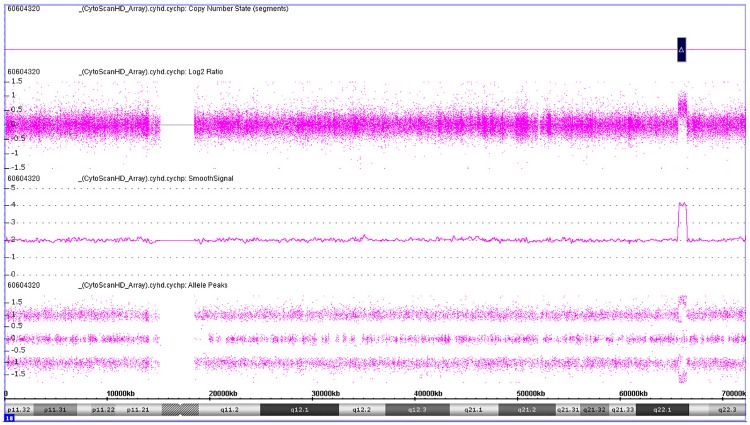
Microarray data for maternal DNA for the patient in Fig 6.

Maternal Microduplications: Early during clinical testing we encountered 2 cases with intermediate Z scores between 3 and 8 (Fig 2). One had a Z score of 5.11 for trisomy 21 and another had a Z score of 6.93 for trisomy 18 (Fig 2). We had recently read a report describing “false-positive” NIPS results due to maternal microduplications [21] and decided to use chromosomal ideograms to investigate whether these intermediate Z scores represented maternal microduplications. Fig 3 shows the ideogram for a typical NIPS result from a fetus confirmed to have trisomy 21. In both of our cases, the ideograms clearly showed that the duplications were in a small portion of the affected chromosomes (Fig 4). With permission from the ordering physicians, we performed microarray analysis on the maternal buffy coat cells, which confirmed the maternal microduplication on chromosome 21 (Fig 5) and 18 (Figs 6 and 7). Henceforth, we examined the ideogram for each chromosome with an elevated Z score before reporting an abnormal result, to ensure the entire chromosome is duplicated and the result is not due to a maternal microduplication. The cumulative data regarding our experiences with maternal microduplications has been submitted elsewhere.

Maternal Global Copy Number Abnormalities: In 1 case, NIPS yielded a positive result for trisomy 21 with a Z score of 21 but amniocentesis revealed a euploid fetus. We reexamined the NIPS data for the entire genome and discovered copy number changes at multiple chromosomes, reflected by elevated Z scores for chromosomes 3, 9, and 21, and negative Z scores (< -8) for chromosomes 4, 6, and 11. The mother had large fibroids [28]. It has been suggested that uterine fibroids can shed DNA into the circulation, causing artificial copy number changes in NIPS analysis [29]. Following this case, we instituted a procedure to examine the entire genome of positive NIPS cases to avoid reporting false-positive results due to global circulating aneuploidy. There have been a total of 6 samples with elevated Z scores for chromosomes 13, 18, or 21 that have also had multiple copy number abnormalities on several other chromosomes. All microarray raw data has been uploaded to: http://www.ncbi.nlm.nih.gov/geo/query/acc.cgi?acc=GSE84810, Accession: GSE84810

### Mosaicism and translocations

There was a single case of Down syndrome with a 14:21 Robertsonian translocation. This case had a highly elevated Z score of 30.78—not unexpected, since most chromosome 21 material was duplicated. Another patient had an intermediate Z score (3.57) for chromosome 21. A second sample, submitted after consulting the physician, had a Z score of 4.22, and a third had a Z score of 5.57. G banding analysis of amniocytes following amniocentesis revealed mosaic trisomy 21 with 7 trisomic cells and 29 euploid cells counted. A case with a highly elevated chromosome 21 Z score (24.43) had amniocentesis demonstrating 15 trisomic cells and 5 euploid cells. A final mosaic case had a Z score of 8.41 for chromosome 21, and fetal mosaicism for Down syndrome was diagnosed by amniocentesis. The amniocyte karyotype was performed by another laboratory, and we could not obtain the ratio. We detected a single mosaic fetus for trisomy 13 following a Z score 10.79. This sample had a trisomy:euploid cell ratio of 16:4.

In all mosaic cases we did not predict the mosaicism from the NIPS analysis. The mosaicism was reported to us when we obtained follow up information on our high risk cases.

Because the percentage of trisomy mosaicism in amniocytes may not reflect the percentage in the chorion, it is difficult to estimate the analytical sensitivity of our assay for mosaic Down syndrome. However, these data suggest that the assay can detect fetuses with as little as 25% trisomic cells.

### Sex chromosome aneuploidies

Maternal genetic variations can also affected sex chromosome aneuploidy screening. In one case positive for 45,X (Turner syndrome), the estimated fetal fraction was >50% and amniocentesis revealed a euploid fetus. Maternal DNA analysis revealed maternal mosaicism for 45,X. Three other cases showed a negative fetal fraction on NIPS; all 3 women were non-mosaic for 47, XXX. Other than the single case of maternal mosaicism for Turner syndrome, all confirmed sex aneuploidies were correctly identified.

### Twins

Four sets of twins had elevated Z scores for trisomy 21. One pregnancy resulted in fetal demise of one twin without genetic testing. In 2 pregnancies, the diagnosis of Down syndrome was confirmed in 1 twin. In a third case, 1 twin had a teratoma and both had normal karyotypes. There was 1 twin gestation positive for trisomy 18, which miscarried without genetic testing.

Unfortunately, we were unable to obtain information on whether these twin gestations were monochorionic or dichorionic. Since 80% of twin gestations are dichorionic we assumed that this was also the case with our series. One might expect Z scores for twins discordant for trisomies to be lower than from singletons, but this does not appear to be case. More data will be necessary before any conclusion can be drawn regarding the mechanism of circulating fetal DNA in twin gestations.

### Positive predictive values

Confirmation of positive NIPS results for trisomy 21 was based on invasive testing. We excluded sonographic findings for trisomy 21 confirmation because most “soft” findings lack specificity. We accepted invasive testing and ultrasound evidence of abnormalities as confirmation of trisomy 18 and 13, since there are clear sonographic findings in both disorders to confirm NIPS results.

In all, 103 pregnancy samples were positive for trisomy 21, including 99 singleton and 4 twin pregnancies. Of these, 87 had successful follow-up; follow-up is pending in 10 patients; and 6 were lost to follow up. Forty-two (48%) of cases with follow-up had confirmation available by invasive testing or physical examination at delivery, including 3 twin gestations (Table 2). The positive NIPS result was confirmed in all but 1 trisomy 21 case, and all twin gestations had 1 affected and 1 unaffected fetus. Thus, the PPV for trisomy 21 was 98%. The single false-positive trisomy 21 NIPS result was associated with multiple maternal genetic abnormalities (described above) and would not have been reported positive using our new reporting criteria. Therefore, with our current practices in place, the PPV for trisomy 21 would have been 100%. Because many pregnancies have no confirmation available, these data must be considered preliminary. In addition to confirmed cases, 8 pregnancies (7 singletons, 1 twin) positive for trisomy 21 on NIPS suffered spontaneous abortion (Table 2), consistent with an increased spontaneous abortion rate for aneuploid pregnancies. Eleven (13%) women with positive trisomy 21 NIPS results elected to terminate their pregnancies without confirmation by invasive testing, while 26 (30%) continued their pregnancies without invasive testing.

Of the 35 singleton and 1 twin pregnancy positive for trisomy 18, 30 had successful follow-up. Direct (invasive testing) or indirect (suspected based on ultrasound findings) confirmation of positive results was available for 25 cases (83%). All but 2 were confirmed to have trisomy 18, yielding a PPV of 92% (Table 2). One false-positive result involved the twin gestation in which 1 twin had a coccygeal mass thought to be a teratoma (described above). This case should have been excluded from NIPS given the frequent chromosomal abnormalities associated with neoplasias. Without this case, the PPV for trisomy 18 would have been 96%. Four (13%) women with positive trisomy 18 NIPS results declined further testing and are continuing their pregnancies. There was only 1 spontaneous abortion among pregnancies positive for trisomy 18.

Twenty-one samples were positive for trisomy 13, including 17 (81%) with complete follow-up: 9 were confirmed positive based on invasive testing or suspected positive based on ultrasound findings, and 4 were false-positives. Thus, the PPV for trisomy 13 was 69%. Of the 4 false-positive cases, 1 involved uterine fibroids (described above; the others remain unexplained. We hope to obtain placental material to investigate the possibility of confined placental mosaicism. These cases could represent vanishing twins or confined placental mosaicism, since they had high Z scores and no global abnormalities.

### Sex chromosome aneuploidies and microdeletions

Of 9 samples positive for Turner syndrome (45,X) (Table 2), 7 had available follow-up data; 1 was false-positive (PPV = 86%). This was the case of maternal mosaicism for Turner syndrome described above. Using our present reporting rules, this case would have been reported as suspected maternal variation because the fetal fraction was >50%. Excluding this would lead to a theoretical 100% PPV for Turner syndrome.

**Fig 8:**
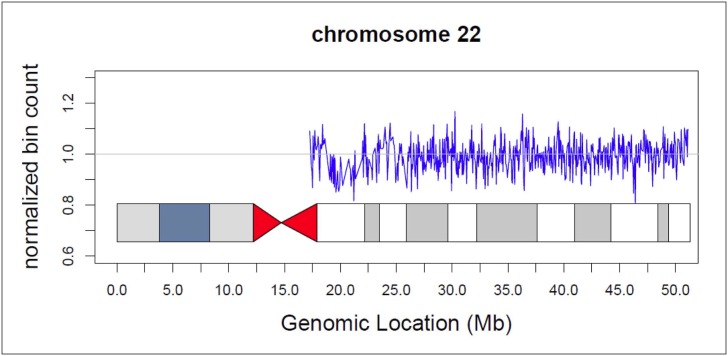
Ideogram for chromosome 22 from a patient with a fetal microdeletion in the DiGeorge region.

Of 5 cases positive for 47,XXX, 2 have follow-up information; both were confirmed to have that karyotype. Two cases were positive for Klinefelter syndrome, and the single fetal genotype we obtained confirmed the 47,XXY karyotype. Only one sample was positive for 47,XYY, but follow-up information was unavailable. We had only one case involving microdeletion in the DiGeorge region of chromosome 22 (Fig 8). The DiGeorge-specific Z score was -7. Amniocentesis confirmed the abnormality. Two samples had 2 abnormalities: 1 with trisomy 21 and Turner syndrome that miscarried and the other with high risk for both trisomy 21 and 18, for which we have not received follow-up data.

## Discussion

We developed a second-generation NIPS test (QNatal Advanced^™^) utilizing technical advancements to increase separation of affected from unaffected pregnancies, along with methods to detect confounding variables such as maternal microduplications and global maternal copy number variation. These refinements were designed to increase the PPV of our NIPS by minimizing false-positive results due to technical issues of “gray zone” indeterminate testing caused by overlap of affected and unaffected pregnancies. With these refinements, the NIPS assay yielded excellent performance characteristics in validation studies and high PPVs for trisomy 21 18, and 13 in clinical practice.

A major strength of this assay is wide separation of Z score thresholds for affected and unaffected pregnancies. In validation studies, no affected pregnancy had a Z score <8 and no unaffected pregnancy had a Z score >4. This clear discrimination allowed us to investigate cases with Z scores between 3 and 8 and has led to detection of 2 important sources of false-positives: maternal microduplications and global copy number abnormalities. We could also detect fetuses with mosaic trisomy 21 and 18. We have implemented procedures that allow detection of microduplications and global abnormalities, which could cause false-positive NIPS analysis in other systems. Although maternal microduplications and global abnormalities were relatively rare (25/10,000; 0.25%), eliminating them as a cause of false-positive results would have a large impact on the PPV of an NIPS test because the prevalence of aneuploid fetuses is <1%. For example, we had 103 samples positive for trisomy 21, including 87 with complete follow up. Using our current reporting rules, the PPV would have been 100%. However, if the single case of multiple maternal abnormalities and the 3 cases of maternal microduplications were counted as false-positive, the PPV would have been only 89%. Similarly, failure to account for these phenomena would have reduced the PPV for trisomy 18 from 100% to 84%, and would have resulted in one-third of positive trisomy 13 results being false positives. Thus, accounting for maternal microduplications and global abnormalities has the potential to ameliorate the relatively high false-positive rates reported for NIPS for trisomy 13 and 18 [30], as well as 21.

Despite the high PPV of this assay, NIPS remains a screening assay. In our clinical follow-up, 10% of women with positive NIPS results for trisomy 21 elected to terminate their pregnancies without confirmation amniocentesis. This finding is in line with a recent report in which 6.2% of women with high-risk NIPS results terminated their pregnancies without invasive follow-up testing [29]. Conversely, nearly one-third of women continued their pregnancies without invasive testing, despite an NIPS test result showing high risk for trisomy 21.

One limitation of this study is the low proportion of samples with confirmation of NIPS results. Although our follow-up numbers remain relatively small, the initial observations are promising in that the observed PPVs are improved over the first-generation NIPS tests. More follow-up data will be necessary to demonstrate this with certainty. We also have no data regarding sensitivities. We are instituting a process of contacting physicians to obtain these data, so hope to be able to determine assay sensitivity in the near future.

Even with our improvements, NIPS must remain a screening test due to both biologic and technical issues that could give rise to false-positive results. As NIPS begins to be offered to low-risk women, the PPV will decline due to the reduced prevalence of the autosomal trisomies. It is distressing that 10% of women terminated their pregnancies following a NIPS report of high risk for trisomy 21 without invasive prenatal diagnostic testing. Clinicians should be encouraged to counsel their patients that, although NIPS is a great advance over maternal serum screening, it remains a screening test.
